# 2,3-Diamino­pyridinium benzoate benzoic acid solvate

**DOI:** 10.1107/S1600536810001443

**Published:** 2010-01-30

**Authors:** Madhukar Hemamalini, Hoong-Kun Fun

**Affiliations:** a X-ray Crystallography Unit, School of Physics, Universiti Sains Malaysia, 11800 USM, Penang, Malaysia

## Abstract

In the title compound, C_5_H_8_N_3_^+^·C_7_H_5_O_2_^−^·C_7_H_6_O_2_, the carboxyl and carboxyl­ate groups are twisted away from their attached benzene rings by 10.75 (7) and 20.33 (6)°, respectively. In the crystal structure, the 2,3-diamino­pyridinium cations, benzoate anions and benzoic acid mol­ecules are linked into a two-dimensional network parallel to (001) by O—H⋯O, N—H⋯O and C—H⋯O hydrogen bonds and π–π inter­actions between the pyridinium rings [centroid–centroid distance = 3.4981 (7) Å].

## Related literature

For substituted pyridines, see: Pozharski *et al.* (1997 ▶); Katritzky *et al.* (1996 ▶). For related structures, see: Fun & Balasubramani (2009 ▶); Balasubramani & Fun (2009*a* ▶,*b* ▶). For bond-length data, see: Allen *et al.* (1987 ▶). For details of hydrogen bonding, see: Jeffrey & Saenger (1991 ▶); Jeffrey (1997 ▶); Scheiner (1997 ▶). For hydrogen-bond motifs, see: Bernstein *et al.* (1995 ▶). For the stability of the temperature controller used in the data collection, see: Cosier & Glazer (1986 ▶).
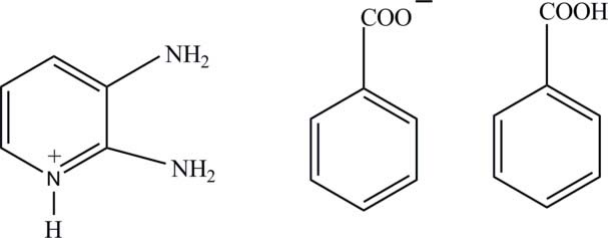


## Experimental

### 

#### Crystal data

C_5_H_8_N_3_^+^·C_7_H_5_O_2_^−^·C_7_H_6_O_2_
*M* *_r_* = 353.37
Monoclinic, 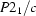

*a* = 12.5822 (2) Å
*b* = 11.0826 (1) Å
*c* = 12.5615 (2) Å
β = 96.345 (1)°
*V* = 1740.89 (4) Å^3^
*Z* = 4
Mo *K*α radiation
μ = 0.10 mm^−1^
*T* = 110 K
0.38 × 0.18 × 0.13 mm

#### Data collection

Bruker SMART APEXII CCD area-detector diffractometer
Absorption correction: multi-scan (*SADABS*; Bruker, 2009 ▶) *T*_min_ = 0.964, *T*_max_ = 0.988
36881 measured reflections
5104 independent reflections
3848 reflections with *I* > 2σ(*I*)
*R*_int_ = 0.037

#### Refinement

*R*[*F* ^2^ > 2σ(*F* ^2^)] = 0.044
*wR*(*F* ^2^) = 0.111
*S* = 1.05
5104 reflections
259 parameters
H atoms treated by a mixture of independent and constrained refinement
Δρ_max_ = 0.32 e Å^−3^
Δρ_min_ = −0.22 e Å^−3^

### 

Data collection: *APEX2* (Bruker, 2009 ▶); cell refinement: *SAINT* (Bruker, 2009 ▶); data reduction: *SAINT*; program(s) used to solve structure: *SHELXS97* (Sheldrick, 2008 ▶); program(s) used to refine structure: *SHELXL97* (Sheldrick, 2008 ▶); molecular graphics: *SHELXTL* (Sheldrick, 2008 ▶); software used to prepare material for publication: *SHELXTL* and *PLATON* (Spek, 2009 ▶).

## Supplementary Material

Crystal structure: contains datablocks global, I. DOI: 10.1107/S1600536810001443/ci5016sup1.cif

Structure factors: contains datablocks I. DOI: 10.1107/S1600536810001443/ci5016Isup2.hkl

Additional supplementary materials:  crystallographic information; 3D view; checkCIF report

## Figures and Tables

**Table 1:** Hydrogen-bond geometry (Å, °)

*D*—H⋯*A*	*D*—H	H⋯*A*	*D*⋯*A*	*D*—H⋯*A*
O1*B*—H1*OB*⋯O1*A*	0.93 (2)	1.66 (2)	2.5796 (13)	173 (2)
N1—H1*N*1⋯O1*A*	0.89 (2)	2.35 (2)	3.0786 (13)	140 (1)
N1—H1*N*1⋯O2*A*	0.89 (2)	2.01 (2)	2.8514 (13)	158 (2)
N2—H1*N*2⋯O2*A*^i^	0.87 (2)	2.07 (2)	2.9370 (14)	173 (2)
N2—H2*N*2⋯O1*A*	0.87 (2)	2.08 (2)	2.9038 (14)	157 (2)
N3—H1*N*3⋯O2*A*^i^	0.88 (2)	2.18 (2)	3.0543 (15)	175 (2)
N3—H2*N*3⋯O1*A*^ii^	0.86 (2)	2.59 (2)	3.0649 (14)	116 (1)
N3—H2*N*3⋯O2*B*^ii^	0.86 (2)	2.16 (2)	2.9912 (15)	162 (1)
C10—H10*A*⋯O2*B*^ii^	0.93	2.58	3.3375 (14)	138

Symmetry codes: (i) 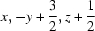; (ii) 
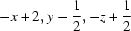
.

**Fig. 1.**
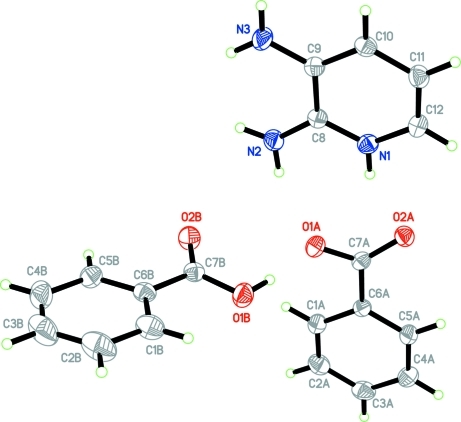
The asymmetric unit of the title compound. Displacement ellipsoids are drawn at the 50% probability level.

**Fig. 2.**
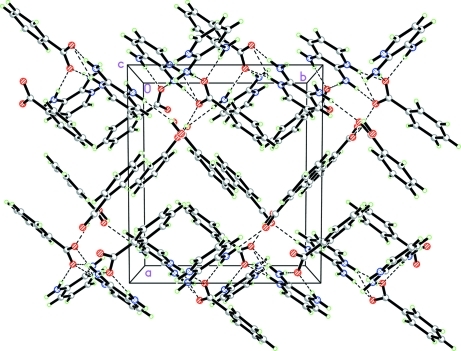
The crystal packing of the title compound, showing hydrogen-bonded (dashed lines) networks.

## supplementary crystallographic information

### Comment

Pyridine and its derivatives play an important role in heterocyclic chemistry (Pozharski *et al.*, 1997; Katritzky *et al.*, 1996). They are often involved in hydrogen-bonding interactions (Jeffrey & Saenger, 1991; Jeffrey, 1997; Scheiner, 1997). Recently, we have reported crystal structures of 2,3-diaminopyridinium 4-hydroxybenzoate (Fun & Balasubramani, 2009), 2,3-diaminopyridinium 4-nitrobenzoate (Balasubramani & Fun, 2009*a*) and 2,3-diaminopyridinium benzoate (Balasubramani & Fun, 2009*b*). In continuation of our studies of pyridinium derivatives, the crystal structure determination of the title compound has been undertaken.

The asymmetric unit of the title compound (Fig. 1), contains a protonated 2,3-diaminopyridinium cation, a benzoate anion and a benzoic acid.  In the 2,3-diaminopyridinium cation, a wide angle (124.11 (11)°) is subtented at the protonated N1 atom. The 2,3-diaminopyridinium cation is planar, with a maximum deviation of 0.010 (1) Å for atom  C10. The carboxyl and carboxylate groups are twisted away from the attached benzene rings; the dihedral angle between C1B–C6B and O1B/O2B/C6B/C7B planes is 10.75 (7)° and that between C1A–C6A and O1A/O2A/C6A/C7A planes is 20.33 (6)°. The bond lengths (Allen *et al.* 1987) and angles are normal.

In the crystal (Fig. 2), the protonated N1 atom and the 2-amino group (N2) are hydrogen-bonded to the carboxylate oxygen atoms (O1A and O2A) via a pair of N—H···O hydrogen bonds forming a *R*_2_^2^(8) ring motif (Bernstein *et al.* 1995). The benzoate anion and benzoic acid molecules are connected via O—H···O hydrogen bonds. The crystal structure is further stabilized by π–π stacking interactions between the pyridinium rings at (x, y, z) and (2-x, 1-y, -z), with a ring centroid-to-centroid distance of 3.4981 (7) Å.

### Experimental

Hot methanolic solution (10 ml) of 2,3-diaminopyridine (27 mg, Aldrich) and a hot aqueous solution  (10 ml) of benzoic acid (31 mg, Merck) were mixed and warmed over a water bath for 10 minutes. The resulting solution was allowed to cool slowly at room temperature.  Single crystals of the title compound appreared from the mother liquor after a few days.

### Refinement

Atoms H1OB, H1N1, H1N2, H2N2, H1N3 and H2N3 were located in a difference Fourier map and refined freely. The remaining H atoms were positioned geometrically [C–H = 0.93 Å] and were refined using a riding model, with *U*_iso_(H) = 1.2*U*_eq_(C).

### Crystal data

**Table:**

C_5_H_8_N_3_^+^·C_7_H_5_O_2_^−^·C_7_H_6_O_2_	*F*(000) = 744
*M**_r_* = 353.37	*D*_x_ = 1.348 Mg m^−^^3^
Monoclinic, *P*2_1_/*c*	Mo *K*α radiation, λ = 0.71073 Å
Hall symbol: -P 2ybc	Cell parameters from 9414 reflections
*a* = 12.5822 (2) Å	θ = 2.5–30.0°
*b* = 11.0826 (1) Å	µ = 0.10 mm^−^^1^
*c* = 12.5615 (2) Å	*T* = 110 K
β = 96.345 (1)°	Block, orange
*V* = 1740.89 (4) Å^3^	0.38 × 0.18 × 0.13 mm
*Z* = 4	

### Data collection

**Table:**

Bruker SMART APEXII CCD area-detector diffractometer	5104 independent reflections
Radiation source: fine-focus sealed tube	3848 reflections with *I* > 2σ(*I*)
graphite	*R*_int_ = 0.037
φ and ω scans	θ_max_ = 30.1°, θ_min_ = 1.6°
Absorption correction: multi-scan (*SADABS*; Bruker, 2009)	*h* = −17→16
*T*_min_ = 0.964, *T*_max_ = 0.988	*k* = −15→15
36881 measured reflections	*l* = −17→17

### Refinement

**Table:**

Refinement on *F*^2^	Primary atom site location: structure-invariant direct methods
Least-squares matrix: full	Secondary atom site location: difference Fourier map
*R*[*F*^2^ > 2σ(*F*^2^)] = 0.044	Hydrogen site location: inferred from neighbouring sites
*wR*(*F*^2^) = 0.111	H atoms treated by a mixture of independent and constrained refinement
*S* = 1.05	*w* = 1/[σ^2^(*F*_o_^2^) + (0.0464*P*)^2^ + 0.4367*P*] where *P* = (*F*_o_^2^ + 2*F*_c_^2^)/3
5104 reflections	(Δ/σ)_max_ = 0.001
259 parameters	Δρ_max_ = 0.32 e Å^−^^3^
0 restraints	Δρ_min_ = −0.22 e Å^−^^3^

### Special details

**Table:**

Experimental. The crystal was placed in the cold stream of an Oxford Cryosystems Cobra open-flow nitrogen cryostat (Cosier & Glazer, 1986) operating at 100.0 (1) k.
Geometry. All esds (except the esd in the dihedral angle between two l.s. planes) are estimated using the full covariance matrix. The cell esds are taken into account individually in the estimation of esds in distances, angles and torsion angles; correlations between esds in cell parameters are only used when they are defined by crystal symmetry. An approximate (isotropic) treatment of cell esds is used for estimating esds involving l.s. planes.
Refinement. Refinement of F^2^ against ALL reflections. The weighted R-factor wR and goodness of fit S are based on F^2^, conventional R-factors R are based on F, with F set to zero for negative F^2^. The threshold expression of F^2^ > 2sigma(F^2^) is used only for calculating R-factors(gt) etc. and is not relevant to the choice of reflections for refinement. R-factors based on F^2^ are statistically about twice as large as those based on F, and R- factors based on ALL data will be even larger.

### Fractional atomic coordinates and isotropic or equivalent isotropic displacement parameters (Å^2^)

**Table:**

	*x*	*y*	*z*	*U*_iso_*/*U*_eq_	
O1A	0.85851 (7)	0.85383 (8)	0.03377 (7)	0.0290 (2)	
O2A	0.95927 (7)	0.88574 (8)	−0.09759 (6)	0.02659 (19)	
C1A	0.74939 (10)	1.07373 (11)	−0.01140 (9)	0.0281 (3)	
H1AA	0.7494	1.0434	0.0576	0.034*	
C2A	0.68601 (11)	1.17233 (12)	−0.04363 (11)	0.0335 (3)	
H2AA	0.6455	1.2097	0.0045	0.040*	
C3A	0.68306 (10)	1.21522 (12)	−0.14752 (11)	0.0336 (3)	
H3AA	0.6393	1.2801	−0.1697	0.040*	
C4A	0.74514 (11)	1.16156 (13)	−0.21788 (10)	0.0348 (3)	
H4AA	0.7426	1.1899	−0.2878	0.042*	
C5A	0.81149 (10)	1.06528 (11)	−0.18501 (9)	0.0274 (3)	
H5AA	0.8549	1.0312	−0.2322	0.033*	
C6A	0.81306 (9)	1.01987 (10)	−0.08187 (8)	0.0214 (2)	
C7A	0.88181 (9)	0.91288 (10)	−0.04745 (8)	0.0222 (2)	
N1	1.05200 (8)	0.68618 (9)	0.02224 (8)	0.0237 (2)	
N2	0.98632 (9)	0.70123 (10)	0.18620 (9)	0.0278 (2)	
N3	1.10673 (10)	0.49546 (11)	0.25569 (9)	0.0336 (3)	
C8	1.04742 (9)	0.64369 (10)	0.12201 (9)	0.0221 (2)	
C9	1.10929 (9)	0.53927 (10)	0.15432 (9)	0.0224 (2)	
C10	1.16824 (9)	0.48674 (10)	0.08034 (9)	0.0244 (2)	
H10A	1.2080	0.4177	0.0992	0.029*	
C11	1.16949 (10)	0.53516 (11)	−0.02261 (9)	0.0268 (3)	
H11A	1.2101	0.4992	−0.0714	0.032*	
C12	1.11068 (10)	0.63515 (11)	−0.05016 (9)	0.0264 (3)	
H12A	1.1106	0.6685	−0.1181	0.032*	
O1B	0.69229 (8)	0.72978 (9)	0.06840 (7)	0.0321 (2)	
O2B	0.75274 (7)	0.77725 (8)	0.23701 (7)	0.0310 (2)	
C1B	0.54529 (11)	0.56665 (13)	0.13724 (12)	0.0364 (3)	
H1BA	0.5578	0.5637	0.0657	0.044*	
C2B	0.46748 (12)	0.49389 (14)	0.17377 (15)	0.0485 (4)	
H2BA	0.4281	0.4417	0.1267	0.058*	
C3B	0.44829 (13)	0.49857 (14)	0.27941 (15)	0.0501 (4)	
H3BA	0.3953	0.4503	0.3033	0.060*	
C4B	0.50734 (12)	0.57449 (14)	0.34983 (13)	0.0443 (4)	
H4BA	0.4943	0.5771	0.4212	0.053*	
C5B	0.58609 (11)	0.64699 (12)	0.31465 (10)	0.0315 (3)	
H5BA	0.6264	0.6975	0.3625	0.038*	
C6B	0.60470 (9)	0.64412 (10)	0.20783 (9)	0.0254 (2)	
C7B	0.69011 (9)	0.72314 (10)	0.17338 (9)	0.0235 (2)	
H1OB	0.7482 (18)	0.7795 (19)	0.0545 (17)	0.076 (6)*	
H1N1	1.0151 (13)	0.7519 (15)	0.0012 (13)	0.038 (4)*	
H1N2	0.9746 (13)	0.6707 (15)	0.2475 (14)	0.041 (4)*	
H2N2	0.9452 (13)	0.7586 (15)	0.1570 (13)	0.045 (5)*	
H1N3	1.0666 (13)	0.5270 (14)	0.3014 (13)	0.040 (4)*	
H2N3	1.1455 (13)	0.4331 (15)	0.2731 (12)	0.038 (4)*	

### Atomic displacement parameters (Å^2^)

**Table:**

	*U*^11^	*U*^22^	*U*^33^	*U*^12^	*U*^13^	*U*^23^
O1A	0.0312 (5)	0.0296 (5)	0.0266 (4)	0.0009 (4)	0.0050 (3)	0.0098 (3)
O2A	0.0266 (4)	0.0268 (4)	0.0270 (4)	0.0029 (3)	0.0057 (3)	0.0008 (3)
C1A	0.0294 (6)	0.0309 (6)	0.0243 (5)	0.0016 (5)	0.0045 (5)	0.0020 (5)
C2A	0.0297 (7)	0.0330 (7)	0.0385 (7)	0.0051 (5)	0.0060 (5)	−0.0029 (5)
C3A	0.0282 (7)	0.0274 (6)	0.0445 (7)	0.0048 (5)	0.0010 (5)	0.0079 (5)
C4A	0.0352 (7)	0.0379 (7)	0.0312 (6)	0.0045 (6)	0.0031 (5)	0.0145 (5)
C5A	0.0282 (6)	0.0299 (6)	0.0246 (5)	0.0014 (5)	0.0055 (5)	0.0051 (4)
C6A	0.0208 (5)	0.0208 (5)	0.0222 (5)	−0.0029 (4)	0.0005 (4)	0.0019 (4)
C7A	0.0241 (6)	0.0219 (5)	0.0200 (5)	−0.0019 (4)	0.0000 (4)	0.0003 (4)
N1	0.0254 (5)	0.0207 (5)	0.0248 (5)	0.0020 (4)	0.0018 (4)	0.0014 (4)
N2	0.0301 (6)	0.0265 (5)	0.0280 (5)	0.0078 (4)	0.0082 (4)	0.0024 (4)
N3	0.0418 (7)	0.0340 (6)	0.0267 (5)	0.0160 (5)	0.0113 (5)	0.0093 (4)
C8	0.0212 (5)	0.0208 (5)	0.0241 (5)	−0.0012 (4)	0.0019 (4)	−0.0005 (4)
C9	0.0221 (6)	0.0214 (5)	0.0235 (5)	−0.0005 (4)	0.0018 (4)	0.0006 (4)
C10	0.0239 (6)	0.0212 (6)	0.0282 (6)	0.0029 (4)	0.0029 (4)	−0.0003 (4)
C11	0.0285 (6)	0.0280 (6)	0.0244 (5)	0.0016 (5)	0.0060 (5)	−0.0029 (4)
C12	0.0302 (6)	0.0279 (6)	0.0213 (5)	−0.0013 (5)	0.0041 (4)	0.0008 (4)
O1B	0.0342 (5)	0.0385 (5)	0.0237 (4)	−0.0063 (4)	0.0036 (4)	−0.0013 (4)
O2B	0.0313 (5)	0.0331 (5)	0.0284 (4)	−0.0075 (4)	0.0031 (4)	−0.0048 (4)
C1B	0.0311 (7)	0.0323 (7)	0.0451 (8)	−0.0034 (6)	0.0011 (6)	−0.0051 (6)
C2B	0.0355 (8)	0.0330 (8)	0.0760 (11)	−0.0094 (6)	0.0020 (8)	−0.0029 (7)
C3B	0.0364 (8)	0.0315 (8)	0.0845 (12)	−0.0028 (6)	0.0156 (8)	0.0184 (8)
C4B	0.0431 (9)	0.0413 (8)	0.0510 (9)	0.0068 (7)	0.0166 (7)	0.0208 (7)
C5B	0.0324 (7)	0.0297 (6)	0.0326 (6)	0.0034 (5)	0.0046 (5)	0.0074 (5)
C6B	0.0230 (6)	0.0207 (5)	0.0321 (6)	0.0033 (5)	0.0017 (5)	0.0017 (4)
C7B	0.0238 (6)	0.0214 (5)	0.0253 (5)	0.0031 (4)	0.0026 (4)	−0.0017 (4)

### Geometric parameters (Å, °)

**Table:**

O1A—C7A	1.2733 (13)	C8—C9	1.4283 (16)
O2A—C7A	1.2541 (14)	C9—C10	1.3802 (16)
C1A—C2A	1.3866 (18)	C10—C11	1.4018 (16)
C1A—C6A	1.3922 (16)	C10—H10A	0.93
C1A—H1AA	0.93	C11—C12	1.3559 (17)
C2A—C3A	1.3855 (19)	C11—H11A	0.93
C2A—H2AA	0.93	C12—H12A	0.93
C3A—C4A	1.3775 (19)	O1B—C7B	1.3241 (14)
C3A—H3AA	0.93	O1B—H1OB	0.93 (2)
C4A—C5A	1.3891 (18)	O2B—C7B	1.2166 (14)
C4A—H4AA	0.93	C1B—C2B	1.385 (2)
C5A—C6A	1.3880 (15)	C1B—C6B	1.3913 (18)
C5A—H5AA	0.93	C1B—H1BA	0.93
C6A—C7A	1.5028 (16)	C2B—C3B	1.376 (2)
N1—C8	1.3461 (14)	C2B—H2BA	0.93
N1—C12	1.3566 (15)	C3B—C4B	1.377 (2)
N1—H1N1	0.888 (17)	C3B—H3BA	0.93
N2—C8	1.3358 (15)	C4B—C5B	1.3855 (19)
N2—H1N2	0.869 (17)	C4B—H4BA	0.93
N2—H2N2	0.875 (17)	C5B—C6B	1.3877 (17)
N3—C9	1.3665 (15)	C5B—H5BA	0.93
N3—H1N3	0.878 (17)	C6B—C7B	1.4873 (17)
N3—H2N3	0.860 (17)		
			
C2A—C1A—C6A	120.34 (11)	N3—C9—C8	118.97 (11)
C2A—C1A—H1AA	119.8	C10—C9—C8	117.84 (10)
C6A—C1A—H1AA	119.8	C9—C10—C11	121.42 (11)
C3A—C2A—C1A	120.00 (12)	C9—C10—H10A	119.3
C3A—C2A—H2AA	120.0	C11—C10—H10A	119.3
C1A—C2A—H2AA	120.0	C12—C11—C10	119.13 (11)
C4A—C3A—C2A	119.88 (12)	C12—C11—H11A	120.4
C4A—C3A—H3AA	120.1	C10—C11—H11A	120.4
C2A—C3A—H3AA	120.1	C11—C12—N1	119.45 (11)
C3A—C4A—C5A	120.38 (12)	C11—C12—H12A	120.3
C3A—C4A—H4AA	119.8	N1—C12—H12A	120.3
C5A—C4A—H4AA	119.8	C7B—O1B—H1OB	108.7 (13)
C6A—C5A—C4A	120.13 (11)	C2B—C1B—C6B	119.86 (14)
C6A—C5A—H5AA	119.9	C2B—C1B—H1BA	120.1
C4A—C5A—H5AA	119.9	C6B—C1B—H1BA	120.1
C5A—C6A—C1A	119.22 (11)	C3B—C2B—C1B	120.22 (15)
C5A—C6A—C7A	120.19 (10)	C3B—C2B—H2BA	119.9
C1A—C6A—C7A	120.58 (10)	C1B—C2B—H2BA	119.9
O2A—C7A—O1A	122.65 (11)	C2B—C3B—C4B	120.18 (14)
O2A—C7A—C6A	119.96 (10)	C2B—C3B—H3BA	119.9
O1A—C7A—C6A	117.38 (10)	C4B—C3B—H3BA	119.9
C8—N1—C12	124.11 (11)	C3B—C4B—C5B	120.23 (15)
C8—N1—H1N1	119.3 (10)	C3B—C4B—H4BA	119.9
C12—N1—H1N1	116.6 (10)	C5B—C4B—H4BA	119.9
C8—N2—H1N2	121.0 (11)	C4B—C5B—C6B	119.88 (13)
C8—N2—H2N2	116.6 (11)	C4B—C5B—H5BA	120.1
H1N2—N2—H2N2	120.2 (15)	C6B—C5B—H5BA	120.1
C9—N3—H1N3	122.8 (10)	C5B—C6B—C1B	119.62 (12)
C9—N3—H2N3	116.8 (10)	C5B—C6B—C7B	118.18 (11)
H1N3—N3—H2N3	120.3 (14)	C1B—C6B—C7B	122.19 (11)
N2—C8—N1	118.79 (11)	O2B—C7B—O1B	122.94 (11)
N2—C8—C9	123.18 (10)	O2B—C7B—C6B	122.38 (11)
N1—C8—C9	118.03 (10)	O1B—C7B—C6B	114.68 (10)
N3—C9—C10	123.18 (11)		
			
C6A—C1A—C2A—C3A	2.2 (2)	N3—C9—C10—C11	−179.41 (12)
C1A—C2A—C3A—C4A	−1.6 (2)	C8—C9—C10—C11	1.28 (17)
C2A—C3A—C4A—C5A	−0.6 (2)	C9—C10—C11—C12	−0.60 (18)
C3A—C4A—C5A—C6A	2.1 (2)	C10—C11—C12—N1	0.01 (18)
C4A—C5A—C6A—C1A	−1.49 (18)	C8—N1—C12—C11	−0.18 (18)
C4A—C5A—C6A—C7A	177.79 (11)	C6B—C1B—C2B—C3B	0.4 (2)
C2A—C1A—C6A—C5A	−0.64 (18)	C1B—C2B—C3B—C4B	−0.9 (2)
C2A—C1A—C6A—C7A	−179.92 (11)	C2B—C3B—C4B—C5B	0.3 (2)
C5A—C6A—C7A—O2A	20.53 (16)	C3B—C4B—C5B—C6B	0.7 (2)
C1A—C6A—C7A—O2A	−160.20 (11)	C4B—C5B—C6B—C1B	−1.17 (19)
C5A—C6A—C7A—O1A	−160.22 (11)	C4B—C5B—C6B—C7B	−179.87 (11)
C1A—C6A—C7A—O1A	19.05 (16)	C2B—C1B—C6B—C5B	0.6 (2)
C12—N1—C8—N2	−179.66 (11)	C2B—C1B—C6B—C7B	179.24 (12)
C12—N1—C8—C9	0.88 (17)	C5B—C6B—C7B—O2B	9.93 (17)
N2—C8—C9—N3	−0.17 (18)	C1B—C6B—C7B—O2B	−168.74 (12)
N1—C8—C9—N3	179.27 (11)	C5B—C6B—C7B—O1B	−170.02 (11)
N2—C8—C9—C10	179.17 (11)	C1B—C6B—C7B—O1B	11.31 (17)
N1—C8—C9—C10	−1.39 (16)		

### Hydrogen-bond geometry (Å, °)

**Table:**

*D*—H···*A*	*D*—H	H···*A*	*D*···*A*	*D*—H···*A*
O1B—H1OB···O1A	0.93 (2)	1.66 (2)	2.5796 (13)	173 (2)
N1—H1N1···O1A	0.89 (2)	2.35 (2)	3.0786 (13)	140 (1)
N1—H1N1···O2A	0.89 (2)	2.01 (2)	2.8514 (13)	158 (2)
N2—H1N2···O2A^i^	0.87 (2)	2.07 (2)	2.9370 (14)	173 (2)
N2—H2N2···O1A	0.87 (2)	2.08 (2)	2.9038 (14)	157 (2)
N3—H1N3···O2A^i^	0.88 (2)	2.18 (2)	3.0543 (15)	175 (2)
N3—H2N3···O1A^ii^	0.86 (2)	2.59 (2)	3.0649 (14)	116 (1)
N3—H2N3···O2B^ii^	0.86 (2)	2.16 (2)	2.9912 (15)	162 (1)
C10—H10A···O2B^ii^	0.93	2.58	3.3375 (14)	138

Symmetry codes: (i) *x*, −*y*+3/2, *z*+1/2; (ii) −*x*+2, *y*−1/2, −*z*+1/2.
